# Football

**Published:** 1892-09-10

**Authors:** 


### FOOTBALL.

The cricket season is past, and that of football has arrived. With its arrival the anxieties of parents commence. It is not desirable to expatiate at length upon the danger and brutality of the game as it is played by Rugby rules. But it is desirable to make constant protests against methods of play which add nothing to the interest of the game whilst they greatly increase its dangers. We are amongst those who have the profoundest conviction of the value of out-door games. Rather than have no such games at all we would even have them a little "brutal." But football, according to Rugby rules, is beyond all reason. If gentlemen would but play like gentlemen, and not like costermongers, the whole football world would soon be brought within the rules of good behaviour and a reasonable degree of safety.

